# Suitability analysis for rice growing sites using a multicriteria evaluation and GIS approach in great Mwea region, Kenya

**DOI:** 10.1186/2193-1801-2-265

**Published:** 2013-06-17

**Authors:** Joseph Kihoro, Njoroge J Bosco, Hunja Murage

**Affiliations:** Jomo Kenyatta University of Agriculture and Technology, 62000 – 00200 Nairobi, Kenya

**Keywords:** Rice production area, Bio-physical Factors, Climatic factors, GIS, Land use variance, Land suitability analysis

## Abstract

Land suitability analysis is a prerequisite to achieving optimum utilization of the available land resources. Lack of knowledge on best combination of factors that suit production of rice has contributed to the low production. The aim of this study was to develop a suitability map for rice crop based on physical and climatic factors of production using a Multi-Criteria Evaluation (MCE) & GIS approach. The study was carried out in Kirinyaga, Embu and Mberee counties in Kenya. Biophysical variables of soil, climate and topography were considered for suitability analysis. All data were stored in ArcGIS 9.3 environment and the factor maps were generated. For MCE, Pairwise Comparison Matrix was applied and the suitable areas for rice crop were generated and graduated. The current land cover map of the area was developed from a scanned survey map of the rice growing areas. According to the present land cover map, the rice cultivated area was 13,369 ha. Finally, we overlaid the land cover map with the suitability map to identify variances between the present and potential land use. The crop-land evaluation results of the present study showed that, 75% of total area currently being used was under highly suitable areas and 25% was under moderately suitable areas. The results showed that the potential area for rice growing is 86,364 ha and out of this only 12% is under rice cultivation. This research provided information at local level that could be used by farmers to select cropping patterns and suitability.

## Introduction

Rice is rapidly becoming a major food in much of sub-Saharan Africa and is set to overtake maize, cassava, sorghum, and other cereals in the near future. The demand is driven as much by population growth as by urbanization. In addition, the high cost of fuel makes rice attractive as it can be prepared quickly and with less energy requirement (Mati and Nyamai 2009). Within Kenya, the demand for rice continues to grow as more Kenyans make changes in their eating habits, and as urban population increases. Rice is currently the third most important cereal crop after maize and wheat. Rice is gaining popularity among the rural folk as well and consumption has risen dramatically over the last three years to stand at 300,000 metric tons per annum. But the annual ranges between 40,000 and 80,000 t. The deficit is met through imports (Mati *et al.*^2011^).

Optimizing rice production can be achieved through sustainable agriculture or farming. The concept of sustainable agriculture or farming involves producing quality products in an environmentally benign, socially acceptable and economically efficient way (Addeo *et al.*^2001^), ensuring optimum utilization of the available natural resource for efficient agricultural production. In order to comply with these principles of sustainable agriculture, one has to grow the crops where they suit best and for which first and the foremost requirement is to carry out land suitability analysis (Nisar Ahamed *et al.*^2000^). Suitability is a function of crop requirements and land characteristics (Mustafa *et al.*^2011^). Matching the land characteristics with the crop requirements gives the suitability. So, ‘Suitability is a measure of how well the qualities of a land unit match the requirements of a particular form of land use’. (FAO 1976). Land suitability analysis has to be carried out in such a way that local needs and conditions are reflected well in the final decisions (Prakash 2003).

Multi-Criteria Evaluation (MCE) approaches and GIS is useful because various production variables can be evaluated and each weighted according to their relative importance on the optimal growth conditions for crops (Perveen *et al.*^2007^). However, the overlay procedure possible in GIS does not enable one to take into account that the underlying variables are not equally important (Janssen and Rietveld 1990). One approach that can help overcome such limitations is MCE, which has received renewed attention within the context of GIS-based decision-making (Pereira and Duckstein 1993). The objective of using MCE models is to find solutions to decision-making problems characterized by multiple alternatives, which can be evaluated by means of decision criteria (Jankowski *et al.*^2001^). In this study, we applied Analytical Hierarchy Process (AHP) in integrating MCE with GIS. The specific objectives of this research were to develop a suitability map for irrigated paddy rice crop (*Oryza sativa*) based on physical and climatic factors of production and to identify potential areas for expanding and optimizing rice production in a rice producing area of Kenya.

## Materials and method

### Study area

The research was carried out in Kirinyaga, Mbeere and Embu counties in Kenya. It is bounded by latitudes 37°13′E and 37°56′E and longitudes 0°10′S and 0°54′S. Annual average precipitation is 950 mm, with the long rains falling between March and May, while the short rainy period is between October and December. The three counties are within the central and Eastern administrative provinces of Kenya. The surface area covers approximately 428,339 hectares.

The area traverses three agro-climatic zones, with maximum moisture availability ratios ranging from 0.65 for zone III toward the highland slopes, to 0.50 for the vast area covered by zone IV, and to 0.4 for the semi-arid zone V (Sombroek *et al.*^1982^). Moisture availability zones are based on the ratio of the measured average annual rainfall to the calculated average annual evaporation. The area is generally hot, with average temperatures ranging between 23 and 25°C, having about 10°C difference between the minimum temperatures in June/July and the maximum temperatures in October/March.

**Figure 1:**
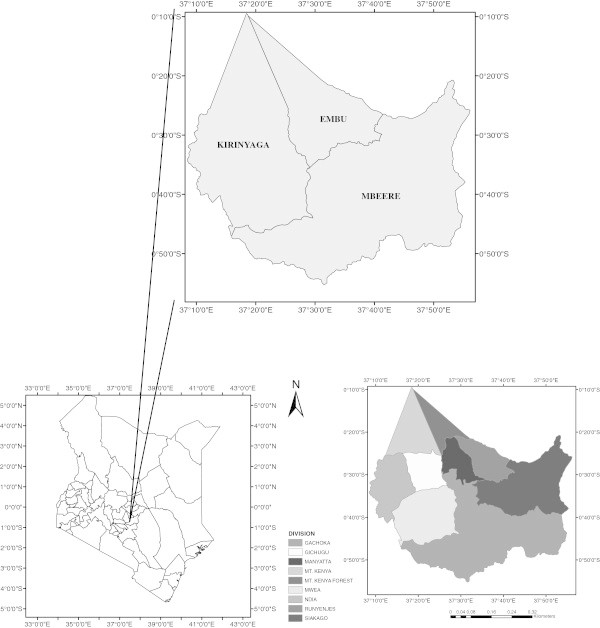
**Location of the study area.**

The predominant soils are vertisols (Sombroek *et al.*^1982^). These are characterized by imperfectly drained clays, very deep, dark gray to black, firm to very firm, and prone to cracking. The most appropriate season for rice cultivation is from August to December, when temperatures are opportune for grain filling and with less risk of disease incidence (Mukiama and Mwangi 1989). However, this period is also when the river flows are at their lowest, coinciding with the dry season, further putting a strain on water available for irrigation (Figure 1).

### Parameters for suitability analysis

Expert opinion of crop specialist was critical in this phase. Literature review of various references, interviews with local agronomists and researchers at Mwea Irrigation and Agricultural Development Centre (MIAD) and desk search of available data helped in identifying the critical requirements for suitable rice growing areas. The factors identified were related to climate (humidity and temperature), soil (soil texture, soil pH, soil drainage) and topography (slope).

**Figure 2:**
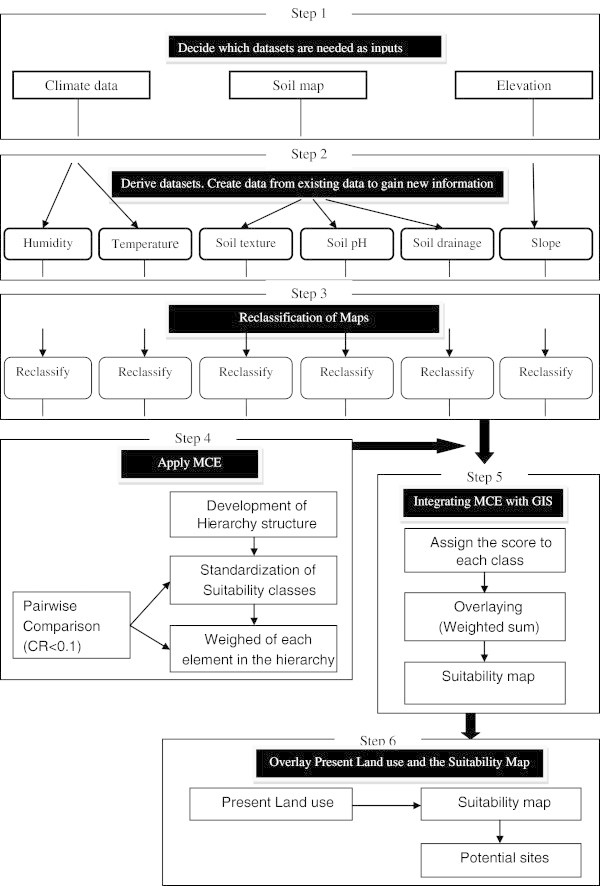
**Flowchart of the methodology followed in the study.**

Climatic information on temperature and humidity was derived from the Exploratory Soil Survey Report (UNEP/GRID 1982) which shows the principle Agro-Climatic Zones of Kenya based on a combination of both moisture availability zones (I-IV) and temperature zones (1–9). Thematic maps were developed for each of the parameters. Data on soil properties was obtained from the Kenya soil survey (KSS). This coverage showed the soil physical and chemical properties of Kenyan soils. The polygons consisted of various soil mapping units linked to an attribute table of soil properties. Three soil parameters of soil texture, soil pH and soil drainage were obtained from an attribute table using Arc GIS 9.3 software and thematic maps were developed for each of the parameters. All the maps were geo-referenced to the Universal Transverse Mercator (UTM) coordinate system. Slope information was obtained from Digital Elevation Model (DEM) using GIS software package ArcGIS 9.3. The source of DEM was Shuttle Radar Topographic Mission (SRTM) which was 90 m spatial resolution. The overall flow chart of the methodology that we followed in this research is illustrated in Figure 2.

### Assigning weight of factors and multi-criteria evaluation (MCE)

**Figure 3:**
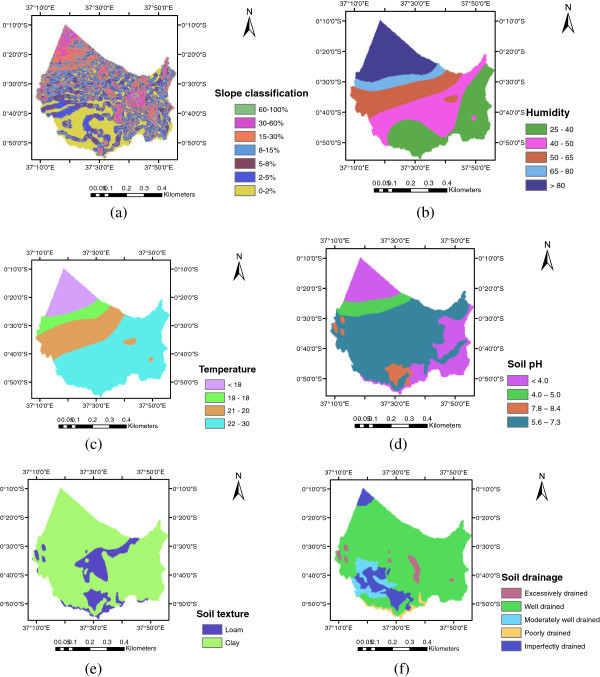
**Suitability levels of the six parameters (a) Slope suitability classes (b) Humidity suitability classes (c) Temperature suitability classes (d) Soil pH suitability classes (e) Soil texture suitability classes (f) Soil drainage suitability classes.**

The purpose of weighting is to express the importance or preference of each factor relative to other factor effects on crop yield and growth rate. Factors established were the most relevant. Suitability levels for each of the factors were defined and used as a base to construct the criteria maps (one for each factor) Figure 3. The suitability levels for each factor were ranked as: Highly suitable-S1, Moderately suitable-S2, Marginally suitable-S3, Not suitable-N, based on the structure of FAO land suitability classification. According to the FAO guide line for irrigated rice and local expert’s opinion, a specific suitability level per factor for rice crop (irrigated) was defined Table 1.

**Table 1:** **Suitability levels of the six parameters**

Scale	Topography	Humidity	Temperature	Soil PH	Soil texture	Soil drainage
Very low suitability	60–100%	< 15	< 18	< 4.0	Sand	E-excessively drained
Low suitability	30 – 60%	15 – 25	> 35	> 8.4	Sandy loam	S-somewhat excessively drained
Moderately low suitability	15 – 30%	25 - 40	19 - 18	4.0 – 5.0	Silt loam	V-very poorly drained
Moderate suitability	8 – 15%	40 - 50	34 - 35	7.8 – 8.4	Loam	W-well drained
Moderately high suitability	5 – 8%	50 - 65	21 - 20	5.1 – 5.5	Silty clay	M-moderately well drained
High suitability	2 – 5%	65 - 80	31 - 33	7.4 – 7.8	Clay loam	P- poorly drained
Very high suitability	0 - 2%	> 80	22 - 30	5.6 – 7.3	clay	I-imperfectly drained

In the procedure for MCE using weighted linear combination, it was necessary that the weights sum to 1. The MCE method used (weighted linear combination) requires that all factors must be standardized (Eastman 1999) or transformed into units that can subsequently be compared (Malczewski 1999). In this study, the factor maps were ranked according to Saaty’s underlying scale with values 1 to 7 by discussion with local crop specialist and from literature reviews as shown in Table 2.

**Table 2:** **Seven-point weighing scale for pair-wise comparison**

Description	Scale
very low suitability	1
low suitability	2
moderately low suitability	3
moderate suitability	4
moderately high suitability	5
high suitability	6
very high suitability	7

Using Pairwise Comparison Matrix, factor weights were calculated by comparing two factors together. The PWCM were applied using a scale with values from 9 to 1/9 introduced by Saaty (1980). A rating of 9 indicates that in relation to the column factor, the row factor is more important. On the other hand, a rating of 1/9 indicates that relative to the column factor, the row factor is less important (Mustafa *et al.,*^2011^). In cases where the column and row factors are equally important, they have a rating value of 1. Table 3 shows pairwise comparison matrix for the research.

**Table 3:** **Pair wise comparison matrix of criteria in AHP**

Scale	Topography	Humidity	Temperature	Soil PH	Soil texture	Soil drainage	Weights	Ranking
Topography	**1**	7	1/3	5	1/3	3	0.1843	3
Humidity	1/7	**1**	1/5	1/3	1/5	1/5	0.0355	6
Temperature	3	5	**1**	7	5	5	0.4153	1
Soil PH	1/5	3	1/7	**1**	1/5	1/ 5	0.0497	5
Soil texture	3	5	1/5	5	**1**	1	0.1865	2
Soil drainage	1/3	5	1	5	1	**1**	0.1287	4
CR = 0.08							∑ = 1	

In the diagonal, elements are assigned the value of unity (i.e., when a factor is compared with itself). Since the matrix is symmetrical, only the lower triangular half actually needs to be filled in. The remaining cells are then simply the reciprocals of the lower triangular half (for example, because the rating of Temperature relative to Topography is 3, the rating of Topography relative to Temperature will be 1/3).

It should be noted that for preventing bias through criteria weighting the Consistency Ratio was used12

- Where: λmax: The maximum eigen value
- CI : Consistency Index
- CR : Consistency Ratio
- RI : Random Index
- n: The numbers of criteria or sub-criteria in each pairwise comparison matrix

Once the composite layers and their weights were obtained, the MCE procedure within Arc GIS 9.3 was applied to produce the map of suitable areas. The suitability map for rice crop (Figure 3) was identified by weighted overlay using spatial analyst tools in ArcGIS 9.3.

### Present land use under rice cultivation

For this research, in order to generate the present Land use under rice growing ground survey map of the scheme area and outgrowers main blocks was obtained from MIAD and JICA. The map was scanned and digitized using Arc GIS 9.3. In order to use these types of data in GIS it was necessary to align it with existing geographically referenced data, the map generated and georeferenced to Arc_1960_ UTM_Zone_36N of WGS 1984.

### Overlay present land use/cover and the suitability map

The present land use/land cover map under rice cultivation and the suitability map for rice crop were overlaid to identify differences as well as similarities between the present land use and the potential land use. For rice crop, a cross table between the map of suitable areas and the land use/land cover map was obtained. In this way, we obtained useful information concerning the spatial distribution of different suitability levels. This phase allowed us to fine-tune our results, because the resultant layer provided the information about how the rice crop was distributed across the various land suitability zones.

## Results and discussions

### Suitability map for rice crop

**Figure 4:**
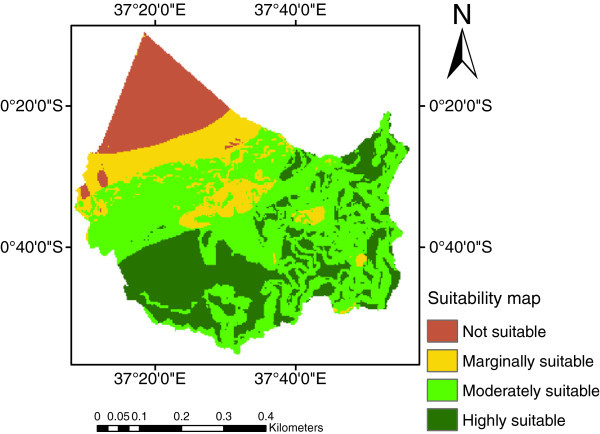
**Rice crop suitability map.**

The suitability map for rice crop, identified by weighted overlay using spatial analyst tools in ArcGIS 9.3, is shown in Figure 4. The number of hectares available to each suitability class was as follows: highly suitable (S1) 105,769 ha, moderately suitable (S2) 203,259 ha, marginally suitable (S3) 61,588 ha and not suitable (N) 57,723 ha which represent 24.69%, 47.45%, 14.39% and 13.48% of land area respectively. The results showed that highly suitable areas (S1) were found mostly in areas under current rice growing. These S1 areas were characterized by: slope level of 0-2%, soil pH level between 5.6 to 7.3, soil drainage imperfectly drained, texture class clay, humidity levels >80 and temperatures between 22-30°C; these values are in agreement with those considered in the literature. Generally not suitable areas (N) were located in mountainous areas with slope level >50%.

According to a related study in the Tana delta, Kuria *et al.* (2011), found the number of hectares available to each suitability class in the Tana delta area to be distributed as follows: 67% is highly to moderately suitable, 14% is moderately suitable, and 10% is marginally suitable. About 9% of the study area classified as Eutric Fluvisol was found to be currently unsuitable for rice cultivation, due to some limitation factors such as partly sandy clay texture, saline, low water retention, and high hydraulic conductivity. Dengiz (2013) did a similar study in Çankırı-Kızılırmak district in the Central Anatolian region of Turkey and found that the land highly and moderately suitable for rice cropping covered an area of about 837.3 ha (55.5%). Of the study area, 34% was unsuitable for rice, and those areas corresponded to adverse soil physical and chemical properties.

### Present land use under rice cultivation

**Figure 5:**
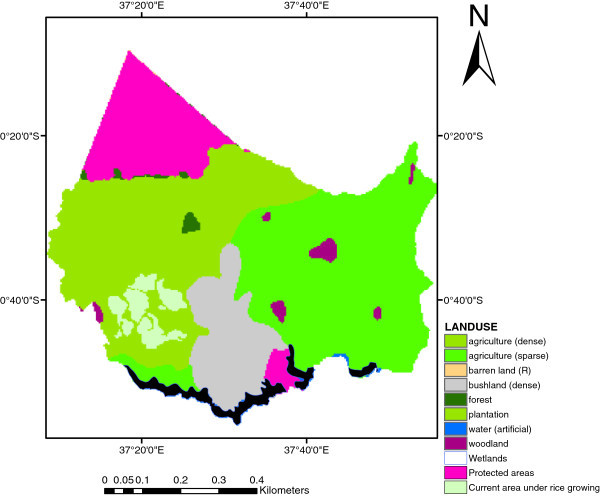
**The current Land use/cover map of the study area.**

Figure 5, shows 10 land use/cover types, within the study area. The rice cultivated area included both the outgrowers blocks and the scheme area. The game reserves and the Mount Kenya forest was classified under protected areas. The total area under rice growing area was 13,369 ha.

### Overlay present land use/cover

**Figure 6:**
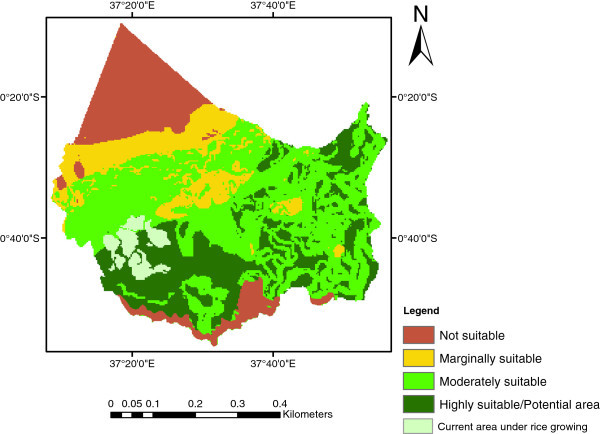
**Potential area map for rice growing.**

To improve the results, the current land use/cover map (Figure 5) and the suitability map for rice (Figure 4) were overlaid to identify differences and similarities between the present land use and the potential land use for the rice crop. This was done because of the identification and accurate description of current and potential production areas are essential for research and agricultural development (Corbett, 1996). The potential area map for rice growing after the overlay is presented in Figure 6. The total potential areas for rice production was 86,364 ha (Table 4). According to the present land use/cover map (Figure 5), the area cultivated with rice was 13,369 ha. The proportion of current rice production areas within the identified suitable areas is shown in Table 5. The analysis revealed that in the study area, 23.08% (3,011 ha) of total rice crop was under Moderately suitable areas and 77.92% (10,036 ha) was under Highly suitable areas. Thus, the average yield of the study area was highly effective since no areas were under the other two classes of marginally and not suitable areas. Therefore, economic levels of agricultural production can be achieved by (a) cultivating rice crop in highly (S1) and moderately (S2) suitable areas, (b) diversification of marginally (S3) suitable areas to crops other than rice that are more suitable in the pedo-climatic requirements.

**Table 4:** **Total potential area for rice growing**

	Areas (Ha)
Suitable area for rice growing	105,769
Area under rice growing	13,369
Potential area for rice growing	86,364

**Table 5:** **Proportion of current rice production areas within the identified suitable areas**

Suitability class	Areas (Ha)	Proportion (%)
Moderately suitable	3,011	23.08
Highly suitable	10,036	76.92

The results of this investigation were adequate in terms of the evaluation criteria set used here because, in a particular project, only a limited number of land qualities need be selected for use in evaluation (FAO 1993). In this investigation, the evaluation criteria were selected taking into consideration the crop requirements regarding local conditions. In this MCE, the factors were selected based on agronomic knowledge of local experts and reviews of existing literature. Such an approach produced valuable information on the relative importance of the factors under evaluation and could be a useful precedent for future studies of rice and other crops. This investigation also provides general alternatives for local farmers in the area of agricultural land management of a particular crop.

## Conclusions and recommendations

In this study, we applied spatial analysis techniques to identify suitable areas for rice crop. The results obtained from this study indicate that the use of GIS and application of Multi-Criteria Evaluation using AHP could provide a superior database and guide map for decision makers considering crop substitution in order to achieve better agricultural production. This approach has been used in some studies in other countries. However, in Kenya this approach is a new and original application in agriculture, because it has not been used to identify suitable areas for rice crop. The study clearly brought out the spatial distribution of rice crop derived from digitizing data in conjunction with evaluation of biophysical variables of soil and topographic information in GIS context is helpful in crop management options for intensification or diversification.

This investigation is a biophysical evaluation that provides information at a local level that could be used by farmers to select their cropping pattern. Additionally, the results of this study could be useful for other investigators who could use these results for diverse studies. For further study, we propose to select more number of factors like soil, climate, irrigation facilities and socio-economic factors which influence the sustainable use of the land.

## Abbreviations

AHP: Analytical Hierarchy Process
GIS: Geographic Information System
MCDA: Multi Criteria Decision Analysis
MCDM: Multi-criteria Decision Making
MCE: Multi-Criteria Evaluation
PWCM: Pairwise Comparison Matrix
SRTM: Shuttle Radar Topographic Mission
UTM: Universal Transverse Mercator.
